# Treatment failure and hospital readmissions in severe COPD exacerbations treated with azithromycin versus placebo – a post-hoc analysis of the BACE randomized controlled trial

**DOI:** 10.1186/s12931-019-1208-6

**Published:** 2019-10-29

**Authors:** Kristina Vermeersch, Ann Belmans, Kris Bogaerts, Iwein Gyselinck, Nina Cardinaels, Maria Gabrovska, Joseph Aumann, Ingel K. Demedts, Jean-Louis Corhay, Eric Marchand, Hans Slabbynck, Christel Haenebalcke, Stefanie Vermeersch, Geert M. Verleden, Thierry Troosters, Vincent Ninane, Guy G. Brusselle, Wim Janssens, On behalf of the BACE trial investigators, Vincent Ninane, Joseph Aumann, Ingel K. Demedts, Hans Slabbynck, Eric Marchand, Christel Haenebalcke, Rudi Peché, Guy G. Brusselle, Walter Vincken, Jean-Louis Corhay, Michiel Haerens, Antoine Fremault, Tine Lauwerier, Alix Debrock, Jan Lamont, Geert Tits, Paul Jordens, Alain Delobbe, Jean-Benoît Martinot

**Affiliations:** 1 Laboratory of Respiratory Diseases, Department of Chronic Diseases, Metabolism and Ageing, KU Leuven, Herestraat 49, O&NI, box 706, B-3000 Leuven, Belgium; 2 Department of Respiratory Diseases, University Hospitals Leuven, B-3000 Leuven, Belgium; 3 I-BioStat, KU Leuven, B-3000 Leuven, Belgium; 4 Universiteit Hasselt, B-3500 Hasselt, Belgium; 5 Department of Pneumology, Centre Hospitalier Universitaire Saint-Pierre, Université Libre de Bruxelles, B-1000 Brussels, Belgium; 6 Department of Pneumology, Jessa Ziekenhuis, B-3500 Hasselt, Belgium; 7 Department of Respiratory Medicine, AZ Delta Roeselare-Menen, B-8800 Roeselare, Belgium; 8 Department of Pneumology, Centre Hospitalier Universitaire, site Sart-Tilman, B-4000 Liège, Belgium; 9 Department of Pneumology, CHU-UCL-Namur, site Mont-Godinne, B-5530 Yvoir, Belgium; 10 Faculty of Medicine, NARILIS, Laboratory of Respiratory Physiology, University of Namur, B-5000 Namur, Belgium; 11 Department of Respiratory Medicine, ZNA Middelheim, B-2020 Antwerpen, Belgium; 12 Department of Pneumology, AZ Sint-Jan, B-8000 Brugge-Oostende, Belgium; 13 Department of Respiratory Medicine, Ghent University Hospital, B-9000 Ghent, Belgium; 14 Department of Rehabilitation Sciences, Faculty of Kinesiology and Rehabilitation Sciences, KU Leuven, Leuven, Belgium

**Keywords:** Recurrent event, Readmission, Macrolide, CRP, Eosinophil count

## Abstract

**Background:**

In the BACE trial, a 3-month (3 m) intervention with azithromycin, initiated at the onset of an infectious COPD exacerbation requiring hospitalization, decreased the rate of a first treatment failure (TF); the composite of treatment intensification (TI), step-up in hospital care (SH) and mortality.

**Objectives:**

(1) To investigate the intervention’s effect on recurrent events, and (2) to identify clinical subgroups most likely to benefit, determined from the incidence rate of TF and hospital readmissions.

**Methods:**

Enrolment criteria included the diagnosis of COPD, a smoking history of ≥10 pack-years and ≥ 1 exacerbation in the previous year. Rate ratio (RR) calculations, subgroup analyses and modelling of continuous variables using splines were based on a Poisson regression model, adjusted for exposure time.

**Results:**

Azithromycin significantly reduced TF by 24% within 3 m (RR = 0.76, 95%CI:0.59;0.97, *p* = 0.031) through a 50% reduction in SH (RR = 0.50, 95%CI:0.30;0.81, *p* = 0.006), which comprised of a 53% reduction in hospital readmissions (RR = 0.47, 95%CI:0.27;0.80; *p* = 0.007). A significant interaction between the intervention, CRP and blood eosinophil count at hospital admission was found, with azithromycin significantly reducing hospital readmissions in patients with high CRP (> 50 mg/L, RR = 0.18, 95%CI:0.05;0.60, *p* = 0.005), or low blood eosinophil count (<300cells/μL, RR = 0.33, 95%CI:0.17;0.64, *p* = 0.001). No differences were observed in treatment response by age, FEV1, CRP or blood eosinophil count in continuous analyses.

**Conclusions:**

This post-hoc analysis of the BACE trial shows that azithromycin initiated at the onset of an infectious COPD exacerbation requiring hospitalization reduces the incidence rate of TF within 3 m by preventing hospital readmissions. In patients with high CRP or low blood eosinophil count at admission this treatment effect was more pronounced, suggesting a potential role for these biomarkers in guiding azithromycin therapy.

**Trial registration:**

ClinicalTrials.gov number. NCT02135354.

## Key questions

### What is the key question?

A 3-month intervention with low-dose azithromycin, initiated at the onset of an infectious COPD exacerbation requiring hospitalization, reduced treatment failure (a novel composite primary endpoint) within 3 months of hospital admission in the BACE trial. The intervention’s effect on the individual exclusive subcomponents of treatment failure and its effect in pre-specified clinical subgroups are not known.

### What is the bottom line?

This comprehensive recurrent-event post-hoc analysis demonstrates that azithromycin significantly reduces the rate of treatment failure within 3 months of hospitalization for an infectious COPD exacerbation by preventing subsequent hospital readmissions with 53%. This treatment benefit is most pronounced in patients with either high CRP (> 50 mg/L) or low blood eosinophil count (< 300 cells/μL) at hospital admission.

### Why read on?

Azithromycin may be a promising intervention to reduce hospital readmissions in a COPD subgroup at high risk. The present findings also suggest a potential role for CRP and blood eosinophil count in guiding azithromycin therapy.

## Introduction

In the field of chronic obstructive pulmonary disease (COPD), acute exacerbations (AECOPD) are considered the most important determinants of a patient’s health status [1–3]. They are characterized by an acute worsening of respiratory symptoms that necessitate additional therapy, based on which they are classified as mild (treated with short-acting bronchodilators only), moderate (requiring systemic corticosteroids and/or antibiotics) or severe (requiring hospitalization) [4]. Hospital readmissions after an initial hospitalization for an exacerbation (‘index event’) are associated with significant morbidity, mortality and high resource utilization [5], especially within the first 3 months after hospital discharge [6].

We recently reported the results of the Belgian trial with Azithromycin for acute COPD Exacerbations requiring hospitalization (BACE, NCT02135354) [7]. The BACE trial was an investigator-initiated randomized placebo-controlled trial (RCT) comparing azithromycin (500 mg once a day for 3 days and subsequently administered for 3 months at 250 mg every 2 days) with placebo on a novel composite primary endpoint, treatment failure (TF). Though formally negative on the primary endpoint (*p* = 0.0526), the applied time-to-first event analyses of its 3 subcomponents revealed that the reduction in TF rate within 3 months of hospital admission was mainly driven by a significant decrease of treatment intensification with systemic corticosteroids and/or antibiotics (TI), as well as step-up in hospital care (SH) for respiratory reasons, while no difference was observed for all-cause mortality. The main analyses, however, limit our understanding to ‘whether’ and ‘when’ a first TF, or one of its subcomponents, occurred [8].

In the present study, we aimed to investigate the intervention’s effect on recurrent events, with a particular focus on hospital readmissions. Based on a number of pre-specified baseline patient characteristics, we additionally aimed to identify clinical subgroups most likely to benefit, determined from the incidence rate of TF and hospital readmissions.

## Methods

The study protocol [9] and primary findings [7] have been published previously.

### The BACE trial

The BACE trial was an investigator-initiated multicentre randomized (1:1) double-blind placebo-controlled trial, evaluating the effectiveness and safety of azithromycin, initiated and uploaded (500 mg once a day for 3 days) within 48-h of hospital admission for a severe infectious AECOPD on top of a standardized acute treatment of systemic corticosteroids and antibiotics (Additional file 1: Table S1), and subsequently administered (250 mg every 2 days) for a prolonged period of 3 months (i.e., 90 days). Patients were followed-up for 6 months thereafter. The study consisted of 3 visits during hospitalization of the index event: randomization (day 1), start of maintenance dose (day 4) and day of discharge (day X, at the investigator’s discretion). After discharge, patients were followed at the out-patient department at 1 month after discharge (day X + 28), end of intervention (day 90) and end of follow-up (day 270). Telephone calls were scheduled bimonthly (day 150 and day 210) between day 90 and day 270.

Patients were monitored for the primary endpoint, TF within 3 months, at day 4, day X, day X + 28 and day 90. TF was defined as the composite of (1) TI with systemic corticosteroids and/or antibiotics for respiratory reasons, (2) SH including transfer to the intensive care unit (ICU) and hospital readmissions for respiratory reasons or (3) all-cause mortality (Additional file 1: Table S2). All patient characteristics were obtained at hospital admission (baseline), except for the spirometry values which were obtained at day X.

The BACE trial was approved by the Commissie Medische Ethiek UZ-KU Leuven (central ethics committee, ML10232). Written informed consent was obtained from all participants.

### Patients

The patient population had an established diagnosis of COPD, a current or past smoking history of ≥10 pack-years and ≥ 1 exacerbation treated with systemic corticosteroids and/or antibiotics in the previous year. The main exclusion criteria were contraindications to azithromycin, respiratory insufficiency requiring mechanical or non-invasive ventilation at the time of randomization, high-dose systemic corticosteroid use (> 4 mg methylprednisolone/day) for ≥2 months and the use of macrolide antibiotics for ≥2 weeks preceding inclusion. A full list of exclusion criteria is provided in Additional file 1: Table S3.

### Statistical analyses

All post-hoc analyses were assessed in the intention-to-treat population (*n* = 301), and were performed using R v3.1.0 (R Core Team, Vienna, Austria) and SAS software v9.4 (SAS Institute, Cary, NC, USA). Count data of TF were analysed by deconstructing TF into exclusive hierarchical subcomponents based on severity (for TF: mortality > SH > TI; for SH: transfer to the ICU > hospital readmission). TI was termed a moderate, and SH and mortality a severe event. The incidence rates and rate ratio’s (RR) were evaluated using a generalized linear model for a Poisson distribution at day X, day 90, and day 270, as well as the post discharge period between day X and day 90, with treatment as factor and log-transformed exposure time as offset. In order to obtain the estimated incidence during 90 and 270 days, the estimated daily rates were multiplied by 90 and 270, respectively. For day X and the post discharge period, the daily rates were multiplied by the median hospital (6 days) and post-hospital (83 days) time. The incidence rates should be interpreted with caution as model assumptions were not entirely met, particularly at day 270.

To assess whether the intervention’s effect on the total number of TFs and hospital readmissions within 3 months varied according to 9 pre-specified baseline patient characteristics, Poisson regression models were used with log-transformed time in the study as offset and treatment, subgroup and their interaction as factors in the model. In these subgroup analyses, TF was defined the composite of the individual subcomponents and hospital readmission included readmissions resulting in ICU transfer or mortality. For CRP and blood eosinophil count at admission, the analysis was additionally stratified for treatment with systemic corticosteroids prior to hospital admission to control for potential confounding [10].

Further analyses were performed for 4 subgroups based on continuous variables (age, CRP, FEV1 and blood eosinophil count), in which the continuous variable was included in the model as a restricted cubic spline to allow for any type of association with the outcome.

All tests were two-sided and assessed at a significance level of 5%. Due to exploratory nature of the analyses, no adjustments were made to the significance level to account for multiple testing.

## Results

### Patients

The present recurrent-event analyses included the intention-to-treat population reported in the primary time-to-first event analyses [7]. The key baseline characteristics of the 301 patients, randomized to azithromycin (*n* = 147) or placebo (*n* = 154), are summarized in Table 1. The extended version is available in the Additional file 1: Table S4. There were no statistically significant group differences.

**Table 1:** Baseline characteristics

	Azithromycin (*N* = 147)	Placebo (*N* = 154)
Demographics		
Age – years	66 ± 9	67 ± 10
Female sex – no. (%)	66 (45)	66 (43)
BMI – kg/m^2^	24.5 ± 5.9	25.1 ± 6.5
Comorbidity		
Charlson comorbidity index	4 [3–5]	4 [3–5]
Lung disease		
mMRC dyspnea score	4 [2–4]	4 [2–4]
Pre-bronchodilator FEV1 – L	0.90 [0.69–1.23]	0.95 [0.71–1.36]
Pre-bronchodilator FEV1 – % pred.	36.0 [26.3–53.8]	38.5 [29.0–52.0]
Pre-bronchodilator FEV1/FVC – %	40.3 [33.6–48.0]	45.0 [37.0–52.8]
GOLD stage – no. (%)^a^		
A	0 (0)	1 (1)
B	26 (18)	30 (20)
C	1 (1)	2 (1)
D	120 (82)	121 (79)
Current smoker – no. (%)	63 (43)	65 (42)
Smoking history – pack-years	44 [37–50]	43 [35–50]
Inhaled therapy for COPD – no. (%)		
LABA	136 (93)	145 (94)
LAMA	118 (80)	123 (80)
Inhaled corticosteroids	118 (80)	123 (80)
SABA	108 (73)	109 (71)
Admission presentation		
GP intervention prior to admission – no. (%)		
Systemic corticosteroids	48 (33)	37 (24)
Antibiotics	50 (34)	54 (35)
Laboratory		
C-reactive protein (mg/L)	14.2 [3.5–61.4]	21.6 [4.5–59.6]
Leucocytes (× 10^9^/L)	10.95 [9.00–13.89]	9.90 [8.20–13.70]
Neutrophils (×10^9^/L)	8.20 [6.00–11.20]	7.70 [5.60–11.20]
Eosinophils (× 10^9^/L)	0.06 [0.00–0.20]	0.07 [0.00–0.20]
Standardized acute treatment		
Received – no. (%)	134 (91)	141 (92)
Received antibiotic – no. (%)	145 (99)	152 (99)
Pathogen susceptible to antibiotic ^b^ − no. (%)	136 (94)	144 (95)

Data are presented as either no. (%), mean ± SD or median [Q1-Q3 interquartile range]

*Abbreviations*: *COPD* Chronic obstructive pulmonary disease, *FEV1* Forced expiratory volume in 1 s, *FVC* Forced vital capacity, *GOLD* Global initiative for chronic Obstructive Lung Disease, guideline 2017, *GP* General practitioner, *LABA* Long-acting beta-agonist, *LAMA* Long-acting muscarinic antagonist, *mMRC* Modified Medical Research Council questionnaire, *SABA* Short-acting beta-agonist

^a^GOLD stages are not taking the current hospital admission into consideration. ^b^Susceptibility was determined based on the need for antibiotic upgrade prior to discharge. Change or narrowing of the initial antibiotic based on proven bacterial cultures was considered good clinical practice

### Recurrent events

**Fig. 1:**
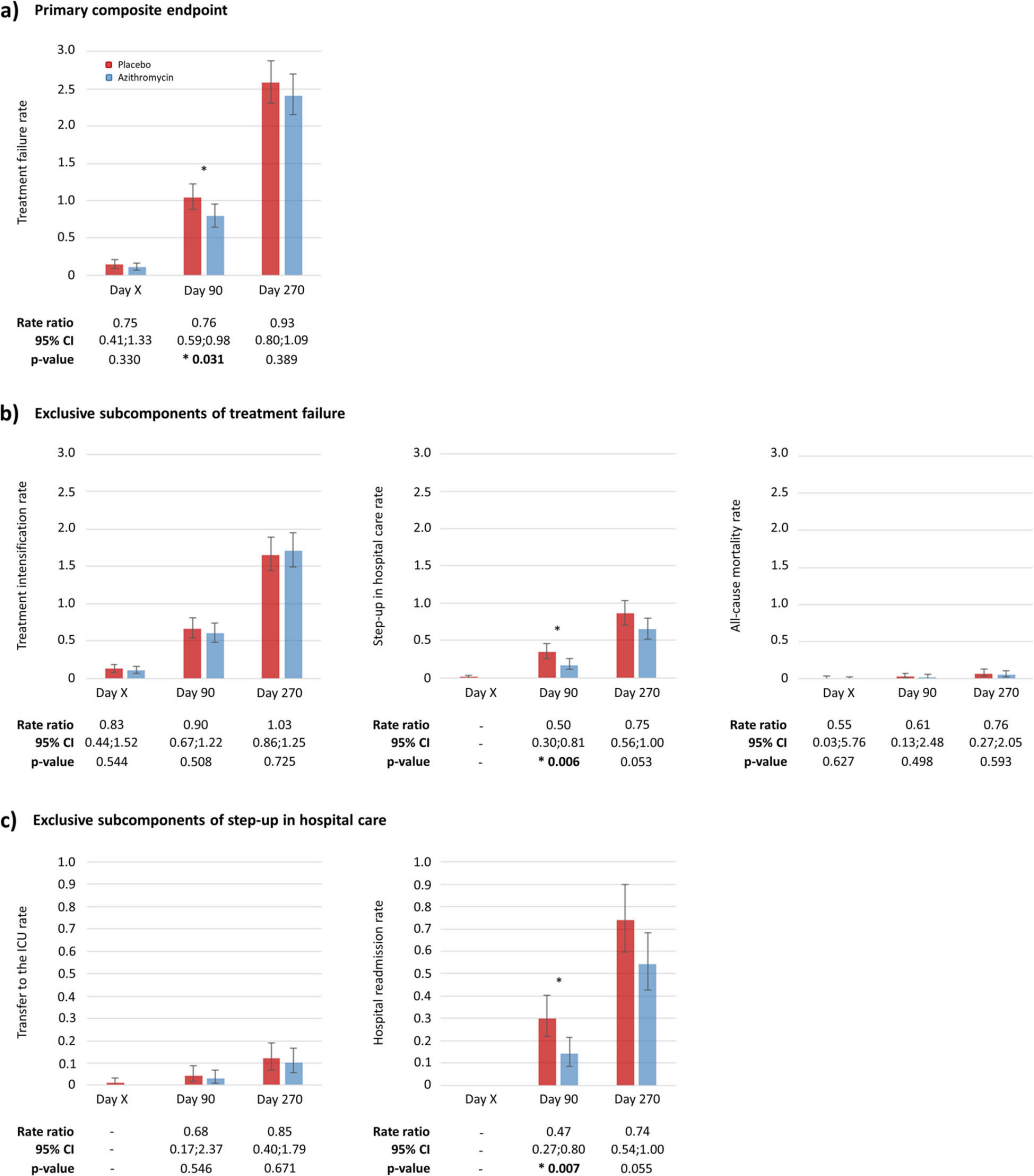
Incidence rate and rate ratio calculations prior to hospital discharge (day X, median: 6 [Q1-Q3 interquartile range: 4–8] days), within 90 and 270 days of **a** the primary composite endpoint, treatment failure, **b** its 3 exclusive subcomponents: treatment intensification, step-up in hospital care and mortality, and **c** step-up in hospital care’s 2 exclusive subcomponents: transfer to the ICU and hospital readmissions. Abbreviations: ICU, intensive care unit. Note: day x, day of discharge, at the investigator’s discretion; day 90, end of intervention; day 270, end of follow-up; *, indicates significant difference

Within 3 months of randomization, a total of 248 TFs occurred: 106 in the azithromycin versus 142 in the placebo group (Table 2), resulting in a significant reduction of 24% (RR = 0.76, 95%CI 0.59;0.98, *p* = 0.031) (Fig. 1a). When decomposing TF in its exclusive hierarchical subcomponents, there was a nominal decrease in all-cause mortality (RR = 0.61, 95%CI 0.13;2.48, *p* = 0.498), a statistically significant reduction of 50% in SH in favour of azithromycin over placebo (RR = 0.50, 95%CI 0.30;0.81, *p* = 0.006) (Fig. 1b) and no difference in TI (RR = 0.90; 95%CI 0.67;1.22, *p* = 0.508). All benefits were lost 6 months after treatment withdrawal, however, SH remained considerably reduced (RR = 0.75, 95% CI 0.56;1.00, *p* = 0.053). 47 TFs (19%) occurred during the hospitalization period of the index AECOPD and were mainly moderate events: 18 in the azithromycin versus 24 in the placebo group (RR = 0.83, 95%CI 0.44;1.52, *p* = 0.544) (Fig. 1b). 201 TFs (81%) occurred during the post-discharge period while on study medication (3-month maximum) and consisted of 64% moderate (*n* = 128; 62 in the azithromycin versus 66 in the placebo group; RR = 0.95 (95%CI 0.67;1.34), *p* = 0.757) and 36% severe events (*n* = 73; 25 in the azithromycin versus 48 in the placebo group; RR = 0.52 (95%CI 0.32;0.84), *p* = 0.009). When assessing the subcomponents of SH within 3 months (Fig. 1c), a non-significant 32% reduction in transfer to the ICU (RR = 0.68, 95% CI 0.17;2.37, *p* = 0.546) was observed, as well as a significant 53% reduction in hospital readmissions (RR = 0.47, 95%CI 0.27;0.80; *p* = 0.007) after hospital discharge of the index AECOPD.

**Table 2:** Count data

	Azithromycin (*n* = 147)			Placebo (*n* = 154)		
a) Number of first events		Day 90	Day 270		Day 90	Day 270
Treatment failure		69	112		86	119
b) Number of recurrent events	Day X	Day 90	Day 270	Day X	Day 90	Day 270
Treatment failure	19	106	306	28	142	322
Treatment intensification	18	80	217	24	90	206
Step-up in hospital care	0	23	82	2	47	107
Transfer to the ICU	0	4	13	2	6	15
Hospital readmission	–	19	69	–	41	92
Mortality	1	3	7	2	5	9

Number of (a) first and (b) recurrent events of the primary composite endpoint, treatment failure, and its 3 exclusive subcomponents: treatment intensification, step-up in hospital care (comprised of transfer to the ICU and hospital readmissions) and mortality prior to hospital discharge (day X, median: 6 [Q1-Q3 interquartile range: 4–8] days), within 90 and 270 days

day x, day of discharge, at the investigator’s discretion; day 90, end of intervention; day 270, end of follow-up

*Abbreviations*: *ICU* Intensive care unit

### Clinical subgroups

Subgroups based on 9 baseline covariates were assessed for the incidence rate of TF and hospital readmissions within 3 months. While no statistically significant interactions were observed between the intervention and any of the subgroups for the incidence rate of TF (Table 3), a significant interaction effect was observed between the intervention and baseline CRP levels (p_interaction_ = 0.0349) for hospital readmissions. Patients with high baseline CRP (> 50 mg/L) treated with azithromycin had significantly fewer hospital readmissions within 3 months, as compared to placebo (RR = 0.18, 95%CI 0.05;0.60, *p* = 0.0053) (Table 4). Furthermore, a significant interaction effect was observed between the intervention and blood eosinophil count at hospital admission (p_interaction_ = 0.0172). Patients with a baseline eosinophil count < 300 cells/μL treated with azithromycin had significantly fewer hospital readmissions within 3 months, as compared to placebo (RR = 0.33, 95%CI 0.17;0.64, *p* = 0.0012) (Table 4). In contrast, patients with a baseline eosinophil count > 300 cells/μL did not show a significant effect on hospital readmission rate when treated with azithromycin. Prior to hospital admission, 28% (*n* = 85) of the randomized patients had received treatment with systemic corticosteroids from their general practitioner. In the non-pretreated patients, the observed interaction between the intervention and both high CRP and low blood eosinophil count for the incidence rate of hospital readmissions remained statistically significant, whereas in the pretreated patients no significant interaction was observed (Table 5).

**Table 3:**
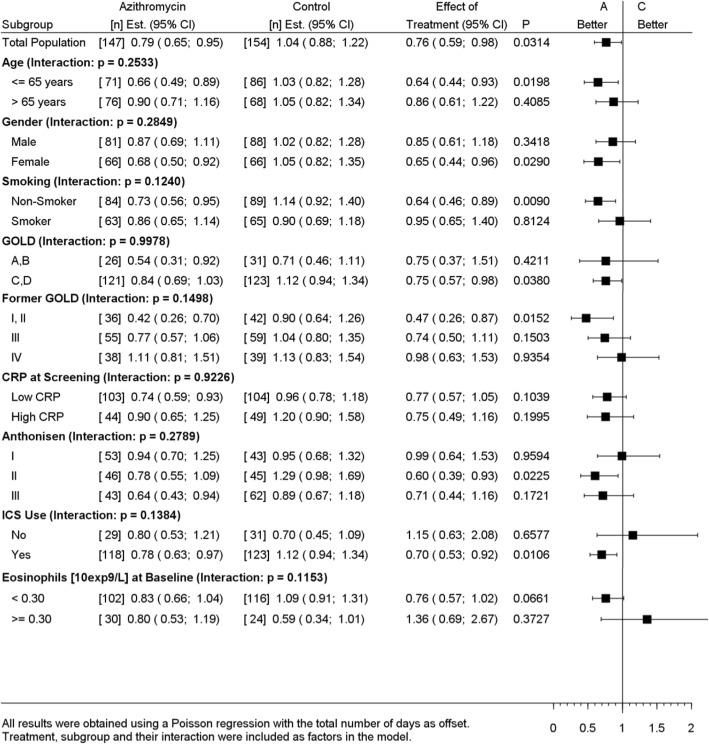
Subgroup analyses of the incidence rate of treatment failure within 90 days, in the intention-to-treat population

low CRP: ≤50 mg/L; high CRP: > 50 mg/L

*Abbreviations*: *CRP* C-reactive protein, *GOLD* Global initiative for chronic Obstructive Lung Disease, guideline 2017, *ICS* Inhaled corticosteroid

**Table 4:**
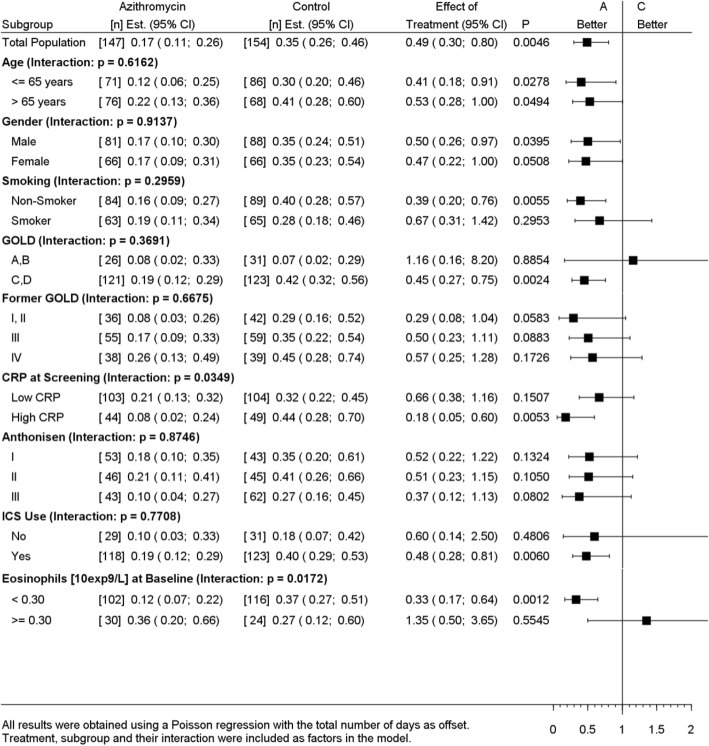
Subgroup analyses of the incidence rate of hospital readmissions within 90 days, in the intention-to-treat population

low CRP: ≤50 mg/L; high CRP: > 50 mg/L

*Abbreviations*: *CRP* C-reactive protein, *GOLD* Global initiative for chronic Obstructive Lung Disease, guideline 2017, *ICS* Inhaled corticosteroid

**Table 5:**
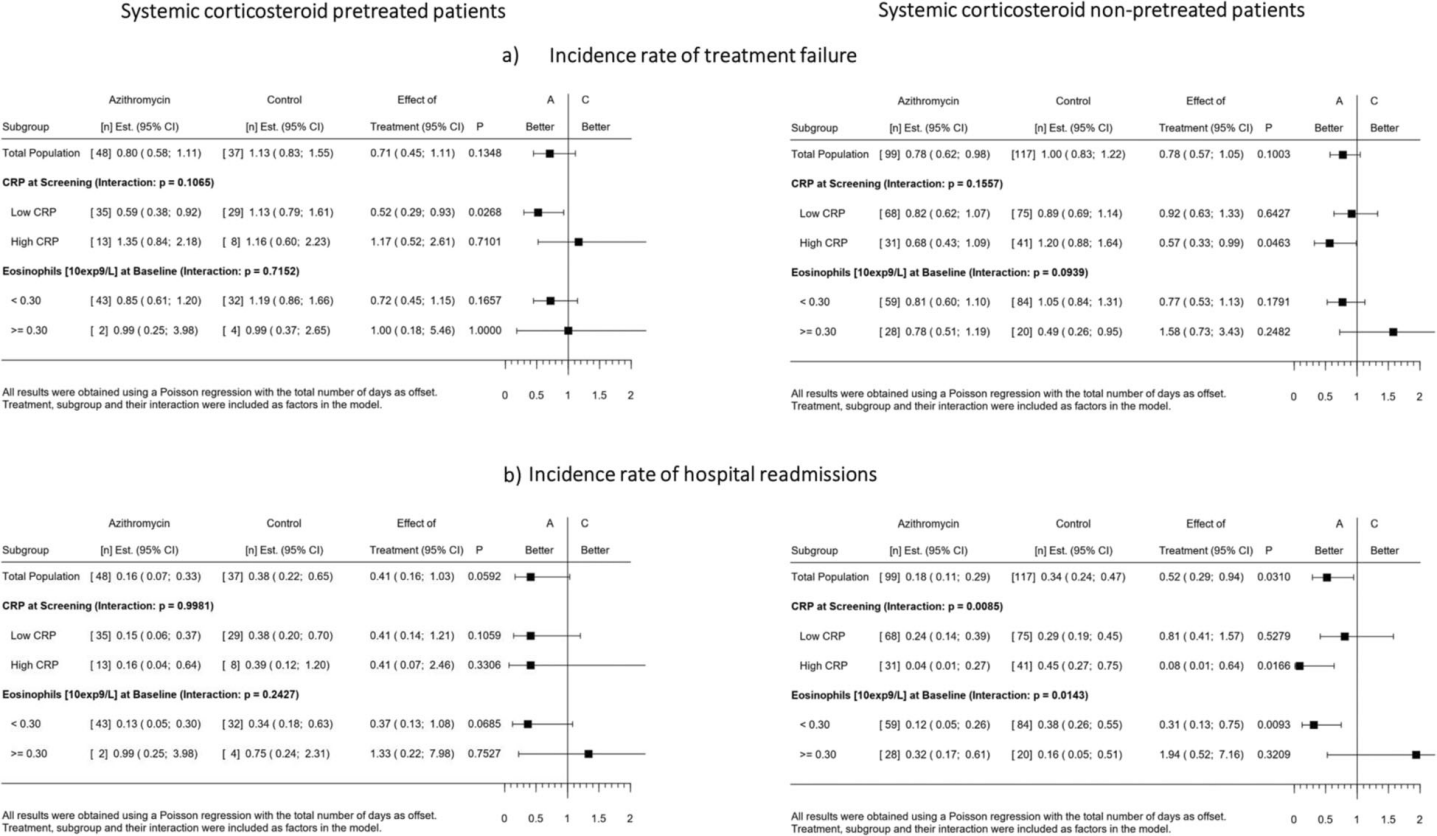
Subgroup analyses of the incidence rate of (a) treatment failure (upper panels) and (b) hospital readmissions (lower panels) within 90 days, in the intention-to-treat population, stratified for treatment with systemic corticosteroids prior to hospital admission

low CRP: ≤50 mg/L; high CRP: > 50 mg/L, pretreated patients (panels left), non-pretreated patients (panels right)

*Abbreviations*: *CRP* C-reactive protein

**Fig. 2:**
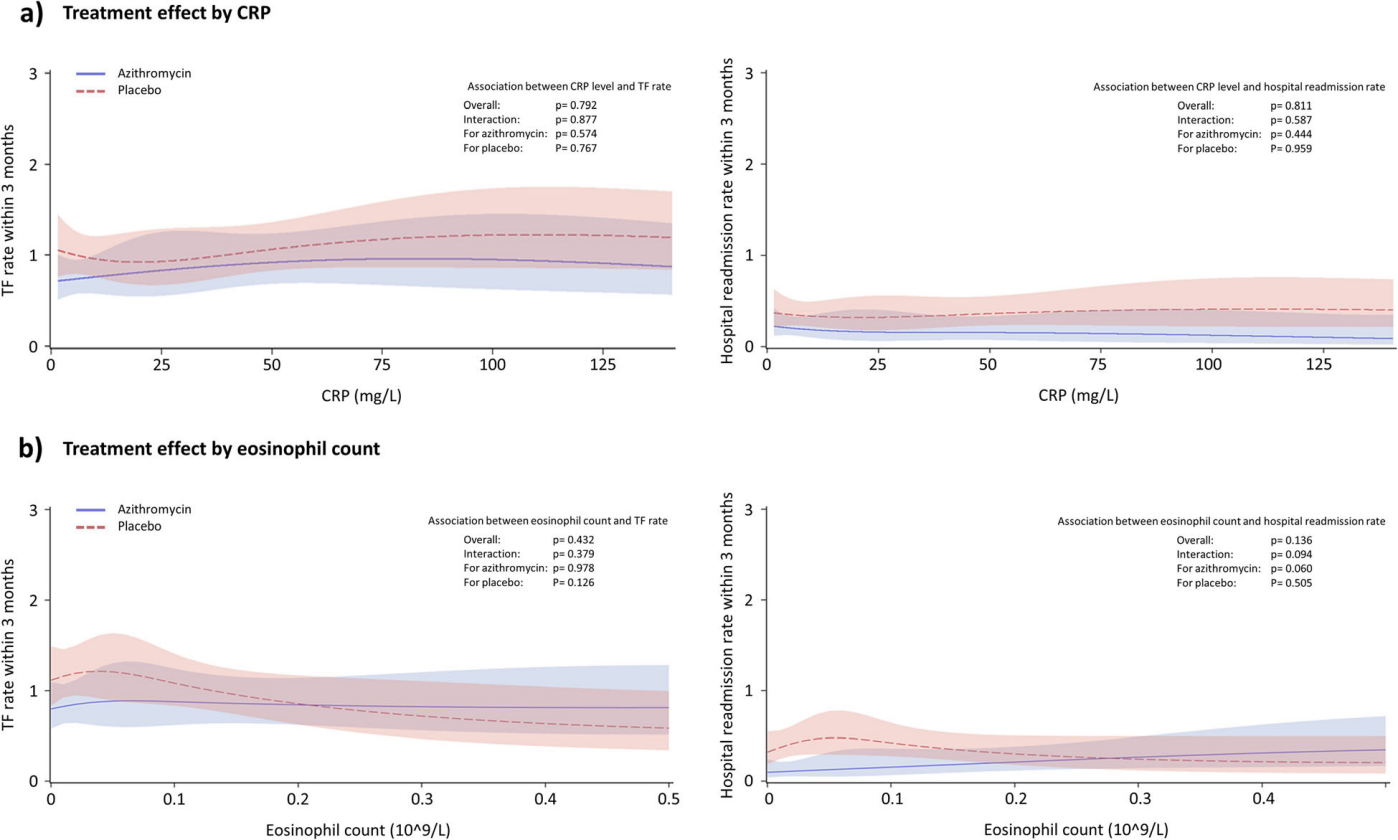
Incidence rate of treatment failure (panels left) and hospital readmissions (panels right) within 3 month by **a** CRP, and **b** blood eosinophil count at day of admission. Abbreviations: CRP, C-reactive protein; TF, treatment failure. Note: Plots are depicted from the 10th to 90th percentile of the respective covariates

From spline modelling, neither age, FEV1 (Additional file 1: Figure S1), CRP nor blood eosinophil count (Fig. 2) were significantly associated with the incidence rate of TF or hospital readmissions. Notably, a *p*-value of 0.094 was observed for the interaction between blood eosinophil count at hospital admission and the incidence rate of hospital readmissions within 3 months.

## Discussion

The BACE trial was an investigator-initiated, randomized, double-blind, placebo-controlled trial evaluating a 3-month intervention with low-dose azithromycin, initiated at the onset of a severe infectious AECOPD requiring hospitalization. Although formally negative on the composite primary endpoint (TF rate within 3 months, *p* = 0.0526), secondary time-to-first event analyses demonstrated a significant 21 and 52% reduction in the rate of TI and SH for respiratory reasons, respectively [7]. The present post-hoc analyses were performed to further elucidate the intervention’s effect on recurrent events, in particular hospital readmissions, and demonstrate that azithromycin reduces the TF rate within 3 months of a severe AECOPD by preventing subsequent hospital readmissions with more than 50%.

COPD is a chronic disease, typically characterized by recurrent exacerbations as the patient’s condition progressively deteriorates [11]. Severe exacerbations requiring hospitalization are the number one contributor to the overall burden and cost of the disease [5], warranting targeted interventions to reduce these readmissions. In the BACE trial, first events accounted for only 63% of the total number of TFs within 3 months. Though conventionally applied, restricting the efficacy analyses to first events would therefore incompletely represent the intervention’s effect on the patient’s overall burden of disease. Moreover, as time-to- first TF was defined as a composite endpoint, with no hierarchy among the different subcomponents, 98% of TFs were first captured by TI even though they were subsequently accompanied by SH or mortality. To provide insight into the true components of TF, the present analyses focus on the exclusive hierarchical subcomponents ranked by severity (mortality > transfer to ICU > hospital readmission > TI with systemic corticosteroids and/or antibiotics). In doing so, we demonstrated that azithromycin reduces the incidence rate of TF at 3 months mainly by reducing subsequent hospital readmissions with 53%, while a clinically relevant though statistically not significant 32% reduction in the transfers to the ICU was also observed. Conversely, we found that azithromycin did not affect the overall incidence rate of moderate events (i.e., treatment intensification not resulting in hospital admission, transfer to ICU or mortality), which – in part – could account for the lack of statistically significant differences in quality of life and symptom assessment scores observed in the BACE trial [7]. When compiled, the BACE trial results substantiate previous findings suggesting azithromycin may be most effective in preventing severe AECOPD requiring hospitalization [12–14]. Azithromycin appears to modify the severity of new exacerbations, so that this high risk subgroup may be less likely to require hospitalization when they do experience new exacerbations. The BACE trial is the first macrolide study in COPD to identify this affordable therapeutic option as a much desired intervention to safely reduce hospital readmissions. Potential adverse effects related to the intervention were discussed previously [7]. No group differences were observed in the number of SH, its exclusive subcomponents (transfer to the ICU and hospital readmissions) or mortality between treatment withdrawal (day 90) and 6 months of follow-up (day 270), supporting earlier time-to-first event analysis findings that prolongation of intake is needed to maintain azithromycin’s clinical benefits. Notably, an increased number of TI in the azithromycin group compared to the placebo group underlies an increase in the number of TF when withdrawing azithromycin.

In the BACE trial, azithromycin was uploaded within 48 h post emergency admission, as potential benefits during the index event were hypothesized to contribute to the overall effect on the primary endpoint. Prior to hospital discharge, azithromycin tended to reduce the incidence rate of TF within 6 days compared to placebo (25% reduction, with a 17 and 45% reduction in TI and all-cause mortality, respectively). These observations were not statistically significant as the study remained largely underpowered for any conclusions on potential benefits during the index event. Several studies, however, have demonstrated that bacterial eradication in the acute phase may result in better long-term outcomes, including readmission rates [15–17]. Future prospective randomized placebo-controlled trials with azithromycin are therefore highly encouraged to evaluate the added value of azithromycin in the acute setting of a severe AECOPD, in addition to a limited prolonged administration to prevent relapse. At this stage, it is up to the clinician to decide when prolonged therapy with macrolides is indicated. Based on the BACE trial findings, patients with COPD suffering from severe infectious exacerbations are at least a target subgroup that needs proper consideration.

The treatment effect of azithromycin appeared to differ according to baseline CRP levels at hospital admission for the index event. Serum CRP has been identified as a relatively sensitive and specific biomarker to distinguish patients who require antibiotics [18, 19], with previous studies showing that a cut-off value of 50 mg/L for high CRP is most optimal to determine antibiotic needs [20–22]. We observed a large and statistically significant effect of azithromycin on hospital readmissions within 3 months in patients with high CRP at baseline (31% of the studied population). This may suggest that the antibacterial effect of azithromycin is the dominant mechanism, although one may argue that all patients were already treated with an effective antibiotic as part of the standardized treatment during the index event. These findings complement the observed 23% significant reduction in the total days of antibiotic use within 3 months in the azithromycin group when compared to placebo (a pre-specified secondary endpoint of the main analysis, *p* < 0.0001) [7]. Similarly, however, azithromycin was found to be more effective in patients with absence of blood eosinophilia at baseline, which corroborates previous findings that high eosinophilia is a good marker for the need of systemic corticosteroids [23]. This observation is biologically plausible, since macrolides have been shown to be effective in neutrophilic chronic airway diseases and might be due to anti-inflammatory and immunomodulatory effects in addition to azithromycin’s antibiotic properties. Although it is tempting to speculate that in AECOPD with high blood eosinophilia azithromycin has no added value, such biomarker guided treatment is needing prospective studies before broad implementation can be recommended. It is currently unknown to what degree azithromycin’s antimicrobial and anti-inflammatory properties contribute to the compiled BACE trial findings. As long as we have no macrolides available that are completely stripped of their antibiotic properties, any further understanding of the mechanism leading to its clinical benefits will be influenced by this dual mode of action.

Through continuous spline modelling, neither age, FEV1, CRP nor blood eosinophil count could be identified as possible predictors of azithromycin response, determined from the incidence rate of TF and hospital readmissions within 3 months. However, we did observe a trend for a negative association between blood eosinophil count at hospital admission for a severe AECOPD and the incidence rate of hospital readmissions within 3 months in patients treated with azithromycin.

The findings of the present study must be interpreted in the context of several potential limitations. Most importantly, this is a post-hoc analysis of a trial which was underpowered for the assessment of the primary endpoint (*p* = 0.0526) due to early termination for slow recruitment. Not observing significant interactions between any of the examined baseline variables and the treatment effect of azithromycin may therefore have been the result of type II errors. Likewise, significant associations obtained from the subgroup analyses may have resulted from an inflated type I error rate, as multiple analyses were performed simultaneously. Furthermore, a cautious interpretation of the findings at day 270 is warranted as not all model assumptions could be met. Prospective and adequately powered clinical trials are therefore needed to verify our hypothesis-generating findings. Second, while TF (as well as each of its subcomponents) allows for a continuous risk exposure during the hospitalization period of the index exacerbation and the post discharge period, a possible difference in exposed risk was not accounted for when calculating the incidence rates. Finally, although studies in COPD have examined thresholds for blood eosinophil counts and CRP levels, it has not yet been defined which measurement – on a continuous rather than a dichotomous scale – is most appropriate to predict treatment response as a biomarker.

## Conclusion

In conclusion, a 3-month intervention with low-dose azithromycin for severe infectious AECOPD requiring hospitalization appears to reduce the rate of treatment failure within 3 months mainly by preventing subsequent hospital readmissions. This complements the BACE trial’s main finding that azithromycin may be a promising and cost-saving therapy for a subgroup of patients with the highest unmet needs. Although rigorous prospective studies are required, the present findings also suggest a potential role for CRP and blood eosinophil count at admission in guiding azithromycin therapy in patients with a severe AECOPD.

## Supplementary information

**Additional file 1: Table S1.** Standardized treatment for an acute COPD exacerbation requiring hospitalization. **Table S2.** Definition of the composite primary endpoint, treatment failure (TF). **Table S3.** Full list of exclusion criteria. **Table S4.** Baseline patient characteristics. **Figure S1.** Incidence rate of TF and hospital readmissions within 3 months by age and pre-bronchodilator FEV1.

## Data Availability

All data generated or analysed during this study are included in this published article, and its supplementary information file.

## Abbreviations

AECOPD: Acute exacerbation of chronic obstructive pulmonary disease
BACE: Belgian trial with azithromycin for COPD exacerbations requiring hospitalization
COPD: Chronic obstructive pulmonary disease
CRP: C-reactive protein
FEV1: Forced expiratory volume in 1 s
ICU: Intensive care unit
RCT: Randomized controlled trial
RR: Rate ratio
SH: Step-up in hospital care
TF: Treatment failure
TI: Treatment intensification
