# Supplementary material for: Treatment failure and hospital readmissions in severe COPD exacerbations treated with azithromycin versus placebo – a post-hoc analysis of the BACE randomized controlled trial
Source: Respir Res. 2019 Oct 29;20:237. doi: 10.1186/s12931-019-1208-6 (PMC6819655; doi:10.1186/s12931-019-1208-6)
Supplement: Supplementary file 1 — Additional file 1: Table S1. Standardized treatment for an acute COPD exacerbation requiring hospitalization. Table S2. Definition of the composite primary endpoint, treatment failure (TF). Table S3. Full list of exclusion criteria. Table S4. Baseline patient characteristics. Figure S1. Incidence rate of TF and hospital readmissions within 3 months by age and pre-bronchodilator FEV1. [file 12931_2019_1208_MOESM1_ESM.docx]

# Additional file 1

**Supplement to:**

Vermeersch K, Belmans A, Bogaerts K, et al. Treatment failure and hospital readmissions in severe COPD exacerbations treated with azithromycin versus placebo – A post-hoc analysis of the BACE randomized controlled trial.

This supplement contains the following items:

**Table S1.** Standardized treatment for an acute COPD exacerbation requiring hospitalization…...………..…1

**Table S2.** Definition of the composite primary endpoint, treatment failure (TF)…………..…………………….….2

**Table S3.** Full list of exclusion criteria………………..………………………………….…………………………………………….…3

**Table S4.** Baseline patient characteristics………………………………….…..…………..……………………………….…….4-5

**Figure S1.** Incidence rate of TF and hospital readmissions within 3 months by age and pre-bronchodilator FEV1…………………………………........……………………………………….…..……………………………………..6

| **Therapy** | **Specifications** |
| --- | --- |
| **Systemic corticosteroids** | Methylprednisolone 40 mg IV or 32 mg PO OD for 5 days  (switch IV to PO as soon as possible) |
| **Antibiotics** |  |
| First choice: | Amoxi-Clavulanate 1 g IV QID or 2 g PO BID for 7 days  (or alternative regimen of 1 g IV QID or 875/125 mg PO TID for 7 days) |
| Alternatives: | Moxifloxacin 400 mg IV or 400 mg PO OD for 5 days |
| In case of: | - Intolerance or allergy to Amoxi-Clavulanate |
|  | - Clinical failure on GP-initiated Amoxi-Clavulanate treatment |
|  | Anti-Pseudomonas antibiotics |
| In case of: | - Bronchiectasis |
|  | - History of positive cultures for Pseudomonas |
|  | - High risk of Pseudomonas |
|  | - Clinical failure on GP-initiated treatment |
| **Short-acting bronchodilators** | Via inhalation |
| **Respiratory support** | Oxygen |
|  | Non-invasive ventilation^a^ |
|  | Mechanical ventilation^a^ |
| **Table S1. Standardized treatment for an acute COPD exacerbation requiring hospitalization Note:** ^a^Considered as exclusion criteria if needed on moment of randomization.  **Abbreviations:** COPD, chronic obstructive pulmonary disease; IV, intravenous; PO, per os; OD, once a day; QID, 4 times a day; BID, 2 times a day; GP, general practitioner | |

| **During hospitalization of the index event** | **After hospital discharge** |
| --- | --- |
| day 1 to day X | day X to day 90 |
|  | |
| **Treatment intensification for respiratory reasons (TI)** | |
| Additional dose of systemic corticosteroids | New course of systemic corticosteroids |
| Prolongation of systemic corticosteroids >8 days | New course of antibiotics |
| Upgrade of antibiotics* |  |
|  | |
|  | |
| **Step-up in hospital care or readmission for respiratory reasons (SH)** | |
| Transfer to the intensive care unit | Readmission |
|  |  |
|  | |
| **All-cause mortality** | |
| **Table S2. Definition of the composite primary endpoint, treatment failure (TF)**  *Change or narrowing of the initial antibiotics given as part of the standardized acute treatment during the index event – *consisting of 5 days of fixed dose systemic corticosteroids and 5 to 7 days of antibiotics* – based on proven bacterial cultures was not considered as treatment failure, but as good clinical practice.  **Note:** day 1: randomization; day X: day of discharge, at the investigator’s discretion; day 90: end of intervention. | |

| **Exclusion criteria** | |
| --- | --- |
| 1 | Mechanical or non-invasive ventilation at the moment of randomization |
| 2 | Prolonged QT interval on ECG: QTcB >450 msec for male or >470 msec for female |
| 3 | History of life-threatening arrhythmias |
| 4 | Myocardial infarction (NSTEMI or STEMI) less than 6 weeks before randomization |
| 5 | Unstable angina pectoris or acute myocardial infarction (NSTEMI or STEMI) at admission |
| 6 | Concomitant use of a drug with high risk for QT prolongation and *Torsade de Pointes*  (amiodarone, flecainide, procainamide, sotalol, droperidol, haldol, citalopram, other macrolides) |
| 7 | Documented uncorrected severe hypokalemia (K^+^ <3.0 mmol/L) or hypomagnesemia  (Mg^2+^ <0.5 mmol/L) |
| 8 | Chronic systemic corticosteroid use (>4 mg methylprednisolone/day for ≥2 months) |
| 9 | Use of macrolides during at least 2 weeks preceding inclusion |
| 10 | Allergy to macrolides |
| 11 | Active cancer treatment |
| 12 | Life expectancy <3 months |
| 13 | Pregnant or breast-feeding subjects. Woman of childbearing potential must have a pregnancy test performed and a negative result must be documented before starting the treatment. |
| **Table S3. Full list of exclusion criteria**  **Abbreviations:** ECG, electrocardiogram; NSTEMI, non-ST elevation myocardial infarction; QTcB, QT interval corrected according to Bazett’s formula; STEMI, ST elevation myocardial infarction | |

|  | **Azithromycin** | | | **Placebo** | | |
| --- | --- | --- | --- | --- | --- | --- |
|  | (N=147) | | | (N=154) | | |
|  |  | | |  | | |
| **Demographics** | | | | | | |
| Age – years | | 66 ± 9 | | | 67 ± 10 | |
| Female sex – no. (%) | | 66 | (45) | | 66 | (43) |
| Weight – kg | | 67 ± 20 | | | 70 ± 18 | |
| Height – m | | 1.66 ± 9 | | | 1.66 ± 9 | |
| BMI – kg/m² | | 24.5 ± 5.9 | | | 25.1 ± 6.5 | |
| **Comorbidity** | | | | | | |
| Charlson comorbidity index | | 4 | [3-5] | | 4 | [3-5] |
| COPD comorbidity index | | 1 | [0-2] | | 1 | [1-2] |
| **Lung disease** | | | | | | |
| mMRC dyspnea score | | 4 | [2-4] | | 4 | [2-4] |
| Pre-bronchodilator FEV1 – L | | 0.90 | [0.69-1.23] | | 0.95 | [0.71-1.36] |
| Pre-bronchodilator FEV1 – % pred. | | 36.0 | [26.3-53.8] | | 38.5 | [29.0-52.0] |
| Pre-bronchodilator FVC – L | | 2.26 | [1.77-3.19] | | 2.24 | [1.80-2.89] |
| Pre-bronchodilator FVC – % pred. | | 73.0 | [58.3-93.8] | | 71.5 | [56.3-88.8] |
| Pre-bronchodilator FEV1/FVC – % | | 40.3 | [33.6-48.0] | | 45.0 | [37.0-52.8] |
| GOLD stage – no. (%)† | |  |  | |  |  |
| A | | 0 | (0) | | 1 | (1) |
| B | | 26 | (18) | | 30 | (20) |
| C | | 1 | (1) | | 2 | (1) |
| D | | 120 | (82) | | 121 | (79) |
| Current smoker – no. (%) | | 63 | (43) | | 65 | (42) |
| Smoking history – pack-years | | 44 | [37-50] | | 43 | [35-50] |
| Number of AECOPD in previous year – no. (%) | |  | | |  | |
| 1 | | 38 | (26) | | 51 | (33) |
| 2 | | 41 | (28) | | 37 | (24) |
| 3 | | 31 | (21) | | 19 | (12) |
| >3 | | 37 | (25) | | 47 | (31) |
| Of which number of hospitalization due to AECOPD – no. (%) | | | | |  | |
| 0 | | 64 | (44) | | 64 | (42) |
| 1 | | 55 | (37) | | 58 | (38) |
| 2 | | 15 | (10) | | 16 | (10) |
| 3 | | 6 | (4) | | 6 | (4) |
| >3 | | 7 | (5) | | 10 | (6) |
| Inhaled therapy for COPD – no. (%) | |  |  | |  |  |
| None | | 5 | (3) | | 5 | (3) |
| ICS only | | 4 | (3) | | 0 | (0) |
| LAMA only | | 2 | (1) | | 3 | (2) |
| LABA only | | 5 | (3) | | 5 | (3) |
| ICS LABA | | 15 | (10) | | 21 | (14) |
| ICS LAMA | | 0 | (0) | | 1 | (1) |
| LAMA LABA | | 17 | (12) | | 18 | (12) |
| ICS LAMA LABA | | 99 | (67) | | 101 | (66) |
|  | |  |  | |  |  |
| Inhaled therapy for COPD, per drug class – no. (%) | |  | | |  | |
| LABA | | 136 | (93) | | 145 | (94) |
| LAMA | | 118 | (80) | | 123 | (80) |
| Inhaled corticosteroids | | 118 | (80) | | 123 | (80) |
| SABA | | 108 | (73) | | 109 | (71) |
| **Admission presentation** | | | | | | |
| Lower respiratory symptoms – no. (%) | |  | | |  | |
| Cough | | 115 | (78) | | 108 | (70) |
| Sputum production | | 97 | (66) | | 86 | (56) |
| Sputum purulence | | 67 | (46) | | 57 | (37) |
| GP intervention prior to admission – no. (%) | |  |  | |  |  |
| Systemic corticosteroids | | 48 | (33) | | 37 | (24) |
| Antibiotics | | 50 | (34) | | 54 | (35) |
| **Laboratory** | | | | | | |
| C-reactive protein (mg/L) | | 14.2 | [3.5-61.4] | | 21.6 | [4.5-59.6] |
| Leucocytes (x10^9^/L) | | 10.95 | [9.00-13.89] | | 9.90 | [8.20-13.70] |
| Neutrophils (x10^9^/L) | | 8.20 | [6.00-11.20] | | 7.70 | [5.60-11.20] |
| Eosinophils (x10^9^/L) | | 0.06 | [0.00-0.20] | | 0.07 | [0.00-0.20] |
| **Standardized acute treatment** | |  |  | |  |  |
| Received – no. (%) | | 134 | (91) | | 141 | (92) |
| Received antibiotic – no. (%) | | 145 | (99) | | 152 | (99) |
| Antibiotic group – no. (%) | |  |  | |  |  |
| ß-lactam antibiotics | | 91 | (62) | | 87 | (57) |
| Quinolone antibiotics | | 61 | (42) | | 71 | (46) |
| Clindamycin | | 1 | (1) | | 1 | (1) |
| Macrolides | | 2 | (1) | | 4 | (3) |
| Pathogen susceptible to antibiotic †† – no. (%) | | 136 | (94) | | 144 | (95) |
|  | |  |  | |  |  |
| **Table S4. Baseline patient characteristics**  Data are presented as either no. (%), mean ± SD or median [Q1-Q3 interquartile range].  **Note:** †GOLD stages are not taking the current hospital admission into consideration. ††Susceptibility was determined based on the need for antibiotic upgrade prior to discharge. Change or narrowing of the initial antibiotic based on proven bacterial cultures was considered good clinical practice.  **Abbreviations:** AECOPD, acute exacerbation of chronic obstructive pulmonary disease; COPD, chronic obstructive pulmonary disease; FEV1, forced expiratory volume in 1 second; FVC, forced vital capacity; GOLD, Global initiative for chronic Obstructive Lung Disease, guideline 2017; GP, general practitioner; LABA, long-acting beta-agonist; LAMA, long-acting muscarinic antagonist; mMRC, modified Medical Research Council questionnaire; SABA, short-acting beta-agonist. | | | | | | |


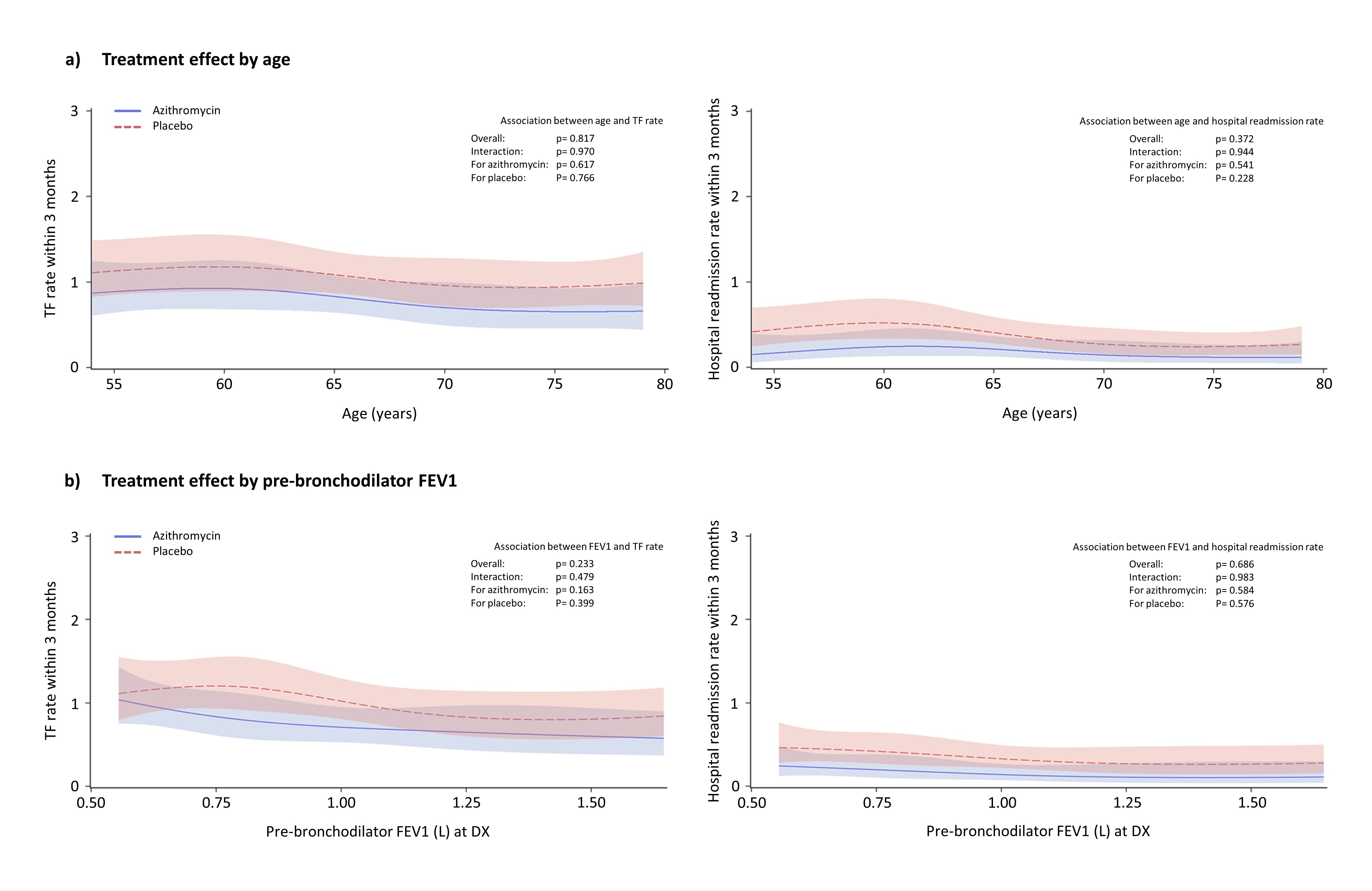


**Figure S1.** Incidence rate of treatment failure (panels left) and hospital readmissions (panels right) within 3 month by (a) age, and (b) pre-bronchodilator FEV1 at day of discharge.

**Abbreviations:** DX, day of discharge; FEV1, forced expiratory volume in 1 second; TF, treatment failure

**Note:** Plots are depicted from the 10^th^ to 90^th^ percentile of the respective covariates.
